# Olive oil polyphenols reduce oxysterols -induced redox imbalance and pro-inflammatory response in intestinal cells

**DOI:** 10.1016/j.redox.2018.05.006

**Published:** 2018-05-16

**Authors:** Gessica Serra, Alessandra Incani, Gabriele Serreli, Laura Porru, M.Paola Melis, Carlo I.G. Tuberoso, Daniela Rossin, Fiorella Biasi, Monica Deiana

**Affiliations:** a Dept. of Food and Nutritional Sciences, University of Reading, RG6 6AP Reading, UK; b Dept. of Biomedical Sciences, Unit of Experimental Pathology, University of Cagliari, 09042 Monserrato, Cagliari, Italy; c Dept. of Life and Environmental Sciences, University of Cagliari, Via Ospedale, 72, 09124 Cagliari, Italy; d Dept. of Clinical and Biological Sciences, University of Turin, 10043 Orbassano, Turin, Italy

**Keywords:** H_2_-DCFH-DA, 2′,7′-dichlorofluorescein diacetate, iNOS, inducible nitric oxide synthase, MAPK, mitogen activated protein kinase, NF-kB, nuclear factor kappa-light-chain-enhancer of activated B cells, NO, nitric oxide, IBD, Inflammatory bowel diseases, NOX-1, NADPH oxidase isoform 1, GSH, reduced gluthatione, COX-2, cyclooxygenase-2, iNOS, inducible nitric oxide synthase, O_2_•^−^, superoxide anion, Olive oil, Polyphenols, Oxysterols, Caco-2 cells, Inflammation, Oxidative stress

## Abstract

Dietary habits may strongly influence intestinal homeostasis. Oxysterols, the oxidized products of cholesterol present in cholesterol-containing foodstuffs, have been shown to exert pro-oxidant and pro-inflammatory effects, altering intestinal epithelial layer and thus contributing to the pathogenesis of human inflammatory bowel diseases and colon cancer. Extra virgin olive oil polyphenols possess antioxidant and anti-inflammatory properties, and concentrate in the intestinal lumen, where may help in preventing intestinal diseases. In the present study we evaluated the ability of an extra virgin olive oil phenolic extract to counteract the pro-oxidant and pro-inflammatory action of a representative mixture of dietary oxysterols in the human colon adenocarcinoma cell line (Caco-2) undergoing full differentiation into enterocyte-like cells. Oxysterols treatment significantly altered differentiated Caco-2 cells redox status, leading to oxidant species production and a decrease of GSH levels, after 1 h exposure, followed by an increase of cytokines production, IL-6 and IL-8, after 24 h. Oxysterol cell treatment also induced after 48 h an increase of NO release, due to the induction of iNOS. Pretreatment with the phenolic extract counteracted oxysterols effects, at least in part by modulating one of the main pathways activated in the cellular response to the action of oxysterols, the MAPK-NF-kB pathway. We demonstrated the ability of the phenolic extract to directly modulate p38 and JNK1/2 phosphorylation and activation of NF-kB, following its inhibitor IkB phosphorylation. The phenolic extract also inhibited iNOS induction, keeping NO concentration at the control level. Our results suggest a protective effect at intestinal level of extra virgin olive oil polyphenols, able to prevent or limit redox unbalance and the onset and progression of chronic intestinal inflammation.

## Highlights

• Dietary oxysterols exerted pro-oxidant and pro-inflammatory effects in differentiated Caco-2 cells.
• H_2_O_2_ production, GSH decrease, IL-6 and IL-8 release were detected.
• NO release due to iNOS induction was higher than controls in oxysterols treated cells.
• Olive oil phenolic extract efficiently counteracted oxysterols effects.
• Phenolic extract directly modulated p38 and JNK MAPK/NF-κB signaling axis.

## Graphical abstract

**Figure:**
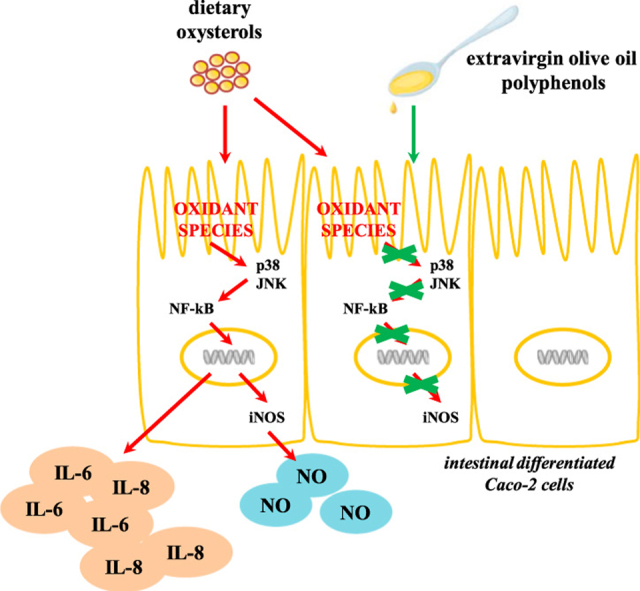

## 1 Introduction

One of the main feature of chronic gastrointestinal inflammatory disorders, such as the inflammatory bowel diseases (IBD), is the overproduction of oxidant species, nitric oxide (NO) and pro-inflammatory cytokines and chemokines, secreted by enterocytes and local immune cells, which sustain and amplify inflammation and cause extensive damage to the mucosa [1], [2]. Growing evidence is accumulating toward the strong influence of dietary components, whose metabolites exert pro-oxidant or pro-inflammatory features, in the onset and progression of gastrointestinal inflammatory disorders [3]. In this connection, dietary oxidized lipids, such as oxysterols and fatty acids hydroperoxides, together with microbiota, may influence intestinal inflammation through different mechanisms which include direct production of reactive species in the colon [4], [5], antigenic effect, alteration of gene expression, changes in the composition of the enteric flora and gut permeability, and immune system deregulation [6].

Oxysterols present in food are the oxidized products of dietary cholesterol and have been reported to reach concentrations ranging from 10 to 100 μM [7], [8]. They have been shown to potentially interfere with the homeostasis of the human digestive tract, by promoting and sustaining irreversible damage and dysfunction of the colonic epithelial barrier, as demonstrated in vitro [9], [10], which may lead to IBD [11] and colon cancer [12], [13]. In previous studies we showed that they may contribute to oxidative imbalance of the intestinal epithelium by inducing the generation of oxidant species [9], [11], at least in part by up-regulating intestinal NADPH oxidase isoform 1 (NOX-1); they are also able to up regulate interleukin (IL)− 8 and IL-6 expression and synthesis [14]. Their pro-inflammatory action seems to be mediated by the modulation of redox-sensible specific mitogen-activated protein kinase (MAPK) signaling pathways, and by activating redox-sensitive transcription nuclear factor kappa B (NF-kB) [15].

Other dietary components, particularly those with antioxidant and anti-inflammatory properties, such as phytochemicals, may be important in preventing or limiting intestinal barrier alterations [3]. Although there is only a very limited number of human trials that have focused on gastrointestinal inflammatory disorders with respect to polyphenols intervention, several in vitro and animal studies have shown the positive effects that polyphenols may play, in particular in the IBD [16]. Few of them concern olive oil polyphenols and show that an extra virgin olive oil diet enriched with phenolic compounds mitigate the severity of DSS-induced colitis in mice, attenuating clinical and histological signs of damage of colonic segments, suppressing oxidative events and inhibiting pro-inflammatory protein expression [17], [18], [19], [20], [21]. The main classes of extra virgin olive oil phenols are phenolic acids, phenolic alcohols, flavonoids, secoiridoids and lignans. Most of them have shown a broad spectrum of antioxidant, free radical scavenger and anti-inflammatory effects [22], which makes them promising dietary supplements in a variety of chronic inflammatory diseases, included IBD [23], [24], [25]. The anti-inflammatory activity of olive oil polyphenols seems to be related to their ability to inhibit the pro-inflammatory activity of oxidants-generating enzymes, including cyclooxygenase-2 (COX-2) and inducible nitric oxide synthase (iNOS) and to modulate different intracellular signaling pathways from NF-kB to MAPKs, through the modulation of redox-sensible cellular networks [23].

However, the signaling pathways related to intestinal inflammation that may be modulated by dietary oxysterols and olive oil polyphenols still need clarifications.

In this contest, our study was aimed to evaluate the ability of an extra virgin olive oil phenolic extract to counteract the pro-oxidant and pro-inflammatory effect of oxysterols in intestinal cells, further exploring the molecular mechanism involved. Human colon adenocarcinoma Caco-2 cell line spontaneously undergoes differentiation into normal enterocyte-like cells, at about 21 days after plating [26]. Differentiated Caco-2 cells were treated with an oxysterol mixture composed by the most widely represented oxysterols in processes or/and stored cholesterol-rich foods [27]. The phenolic extract used, was obtained from a monovarietal extra virgin olive oil of Bosana cultivar, one of the most common and widespread varieties in Sardinia (Italy), extracted trough industrial standard procedures [28]. The protective action of the phenolic extract was evaluated as ability to modulate cellular redox status alteration and MAPKs-NF-kB activation induced by oxysterols and the inflammatory mediators acting downstream of this pathway, IL-6 and IL-8, NO and iNOS.

## 2 Materials and methods

### 2.1 Reagents

Unless otherwise specified, all reagents and chemicals were from Sigma-Aldrich (Milan, Italy). 5-cholesten-3β,7α-diolo (7α-hydroxycholesterol) and 5-cholesten-3β,7β-diolo (7β-hydroxycholesterol) were purchased from Avanti Polar Lipids (Alabaster, Alabama, USA). Cell culture materials were purchased from Invitrogen (Milano, Italy) and from Lonza (Basel, Switzerland). The primary antibodies anti-phospho-JNK 1/2, anti-JNK, anti-phospho-p38, anti-p38 and the enhanced chemiluminescence (ECL) reagent were purchase from Millipore (Watford, UK). Anti-iNOS (C-11), anti-p-IκB (B-9) and anti-IκB (9242) mouse monoclonal primary antibodies where purchased from Santa Cruz Biotechnology (Dallas, Texas, USA). Gels and all material for electrophoresis and immunoblotting were purchased from Invitrogen (Milan, Italy). Nitrocellulose membranes were obtained from Amersham (Little Chalfont, UK).

### 2.2 Cell culture and treatments

Human colon adenocarcinoma Caco-2 cells (ECACC Salisbury, Wiltshire UK) were cultured in Dulbecco's modified Eagle's medium (DMEM), supplemented with 10% heat-inactivated bovine serum, 2 mM L-glutamine, 1% nonessential amino acids, 100 U/ml penicillin, and 100 μg/ml streptomycin, in monolayers at 37 °C in a humidified atmosphere of 5% CO_2_. For experimental studies Caco-2 cells, at passage 45–60, were plated and used 18–21 days post seeding, when fully differentiated. Cells were treated with the oxysterol mixture, composed of 42.96% 7-ketocholesterol, 32.3%, 5α,6α-epoxycholesterol, 5.76% 5β,6β-epoxycholesterol, 4.26% 7α-hydroxycholesterol, and 14.71% 7β-hydroxycholesterol, to a final concentration of 60 μM, at different times to locate the temporal window for investigating the activation of different cell signaling pathways. In a parallel set of experiment, 30 min prior to the oxysterols exposure, cells were treated with different amounts of the phenolic extract in PBS solution (1–25 μg/ml).

### 2.3 Preparation of the olive oil phenolic extract

The phenolic fraction was extracted from a monovarietal extra virgin olive oil obtained from an olive orchard located in South Sardinia (Villasor, Cagliari, Italy). Olive trees were of Bosana cultivar, and the oil extraction was performed in an industrial oil extraction plant following standard procedures by the AGRIS technicians, as previously reported [28]. The separation of the phenolic fraction was carried out through a liquid–liquid extraction method using MeOH/H_2_O 80:20 (v/v) and phenolic components were identified and quantified by LC-DAD analyses as previously described by Incani et al. [28].

### 2.4 Evaluation of IL-8 and IL-6 protein levels

Differentiated Caco-2 cells seeded in 6-well plates (5 ×10^4^ cells/ml in 2 ml) were treated for 24 h with the oxysterol mixture, and pre-treatment with the phenolic extract in a set of samples; at the end of the incubation time the culture medium was collected and used for ELISA detection. Levels of IL-8 and IL-6 were quantified using the Human IL-8 ELISA kit (Campoverde s.r.l., Milano, Italy) and the Human IL-6 ELISA kit (Pantec s.r.l., Torino, Italy) following the manufacturer's instructions. Sample absorbance values were read at 450 nm with a wavelength correction of 550 nm in a microplate reader (Model 680 microplate reader Bio-Rad), and data analyzed using SlideWrite Plus software (Advanced Graphics Software).

### 2.5 Measurement of nitric oxide production

In order to analyze the nitric oxide release, differentiated Caco-2 cells, seeded in 6-well plates (5 ×10^4^ cells/ml in 2 ml), were rinsed with PBS and cultured for 24 h in serum-free, phenol red-free DMEM with L-arginine 0.1 mM. A time course analysis of NO production was performed by incubating cells from 18 to 72 h. In another set of experiments cells were also pre-incubated with phenolic extract before for 30 min and then incubated with oxysterols with further 24 h. At the end of different incubation times NO production was evaluated in terms of quantity of nitrite accumulated in the culture medium by using the Griess’ reagent. Nitrite concentration was determined by mixing 100 μl of the collected medium with an equal volume of Griess’ reagent and incubating at room temperature for 15 min. Absorbance values were read at 540 nm, and nitrite levels were determined with a sodium nitrite standard curve ranging from 0.1 to 10 μM.

### 2.6 Determination of intracellular H_2_O_2_ production

Intracellular H_2_O_2_ production was monitored in differentiated Caco-2 cells seeded in 96-well plates (5 ×10^4^ cells/ml in 100 μl). The old medium was removed, cells were washed with 200 μl of PBS and incubated for 30 min with 2′,7′-dichlorofluorescin diacetate (H_2_-DCFH-DA), 10 μM. H_2_-DCFH-DA was then replaced by the PBS solution containing the phenolic extract (5–25 μg/ml), 30 min prior to adding the oxysterol mixture and incubated for 1 h. Hydrogen peroxide production was monitored by reading the fluorescence emitted, using a micro plate reader (Infinite 200, Tecan, Salzburg, Austria) at a controlled temperature of 37 °C. The reading was performed using an excitation of 490 nm and an emission of 520 nm [29].

### 2.7 Determination of intracellular reduced glutathione (GSH) levels

GSH levels were determined in Caco-2 cells, grown and differentiated in 6-well plates (5 ×10^4^ cells/ml in 2 ml). After preincubation with the phenolic extract, cells were treated with the oxysterol mixture for 1 h.

After treatment cells were scraped into 200 μl of ice-cold PBS and centrifuged at 10000 g for 20 min at 4 °C. The pellet was used to determine GSH levels through EC-HPLC quantification (Agilent 1260 infinity coupled with an electrochemical detector DECADE II Antec, Leyden, Netherlands) [29].

### 2.8 Immunoblotting

All experiments were performed in differentiated Caco-2 cells seeded in 6-well plate (5 ×10^4^ cells/ml in 2 ml). Following treatment, the cells were washed with ice-cold PBS prior to the addition of 180μl CelLytic M lysis buffer for protein extraction, added with mammalian protease and phosphatase inhibitor cocktail (1:100 v/v). Cells were scraped on ice and lysates were incubated for 15 min on ice before centrifugation at 12500 g at 4 °C for 7 min. The protein concentration was determined by the Bradford protein assay [30].

iNOS level evaluation was performed upon immunoprecipitation: total cell extracts (150 μg protein) were immunoprecipitated overnight with mouse anti-iNOS polyclonal antibody (5 μl). Immunoprecipitation was achieved by 2 h incubation at room temperature with Protein A-Sepharose resin, and pellets were used for immunoblotting.

The samples were boiled at 90 °C for 10 min in boiling buffer (62.5 mM Tris, pH 6.8 containing 2% SDS, 5% 2-mercaptoethanol, 10% glycerol, and 0.0025% bromophenol blue). The boiled samples were run on 4–12% SDS–polyacrylamide gels (20 lg/lane), and the proteins were transferred to nitrocellulose membranes (Hybond-ECL) by eBlot Protein Transfer Device with a 7–9 min transfer setup (Twinhelix, Milan, Italy).

The nitrocellulose membrane was then incubated in a blocking buffer (TBS) containing 4% (w/v) skimmed milk powder for 30 min at room temperature, followed by two 10 min washes in TTBS. The blots were then incubated with anti-pp38 (1:1000 dilution), anti-p38 (1/1000 dilution), anti-pJNK 1/2 (1:1000 dilution), anti-JNK 1/2 (1:1000 dilution), anti-iNOS (1:500 dilution), anti-IκB (1:1000 dilution) and anti-p-IκB (1:1000 dilution), in TTBS containing 1% (w/v) skimmed milk powder (antibody buffer) overnight at 4 °C on a three-dimensional rocking table. The blots were washed twice for 10 min in TTBS and then incubated with goat anti-rabbit and anti-mouse IgG conjugated to horseradish peroxidase (1:2000 or 1:5000 dilution) in antibody buffer for 45 min. Finally, the blots were washed twice for 10 min in TTBS, exposed to Clarity™ Western ECL for 2–5 min and developed with the ChemiDoc™ XRS+ Imager (BioRad Laboratories, Inc., Hercules, California, USA). The molecular weights of the bands were calculated from comparison with pre-stained molecular weight markers that were run in parallel with the samples (range 14–180 kDa, GenScript, Piscataway, NJ). Protein bands were quantified using Quantity One software (BioRad Laboratories).

### 2.9 Statistical analyses

Results are expressed as means ± standard deviations. The statistical significance of parametric differences among sets of experimental data was evaluated by the one-way ANOVA test associated with Bonferroni's multiple comparison post test using GraphPad InStat (GraphPad Software, San Diego, Calif., U.S.A.).

## 3 Results

### 3.1 Olive oil phenolic extract inhibits oxysterols-induced IL-8, IL-6 and NO production

The ability of the phenolic extract to modulate the oxysterols-induced pro-inflammatory effect in Caco-2 cells was evaluated measuring the production of IL-8, IL-6 and NO.

**Fig. 1:**
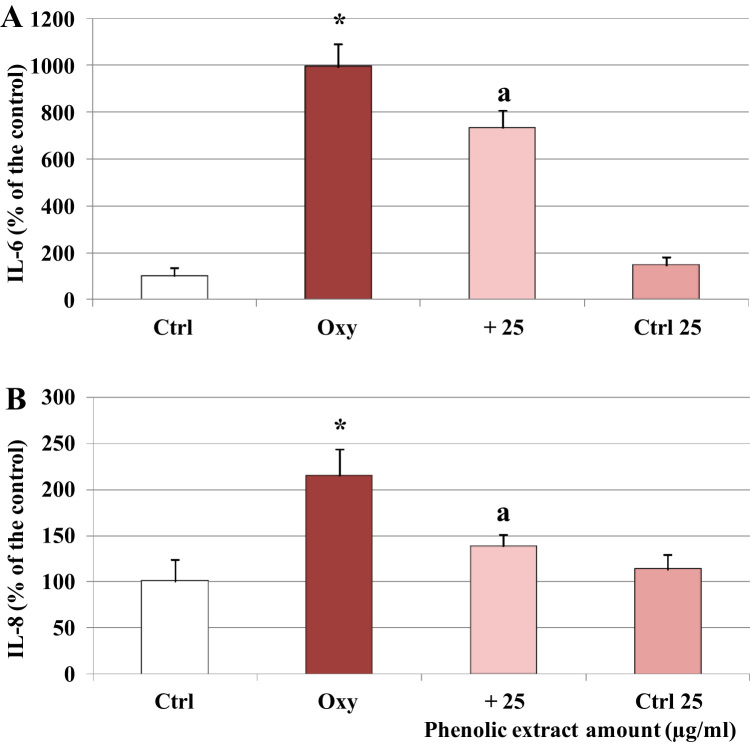
Modulation of IL-8 and IL-6 protein levels by oxysterols and phenolic extract. Interleukins levels, IL-6 (A) and IL-8 (B), in differentiated Caco-2 cells pretreated or not with the phenolic extract (25 μg/ml) for 30 min and incubated for 24 h in presence of 60 μM oxysterol mixture. Interleukins levels were also evaluated in cells treated only with the phenolic extract (Ctrl 25). Values, expressed as percentage of the controls (control values were IL-8 = 6.23 pg/ml, IL-6 = 2.87 pg/ml), represent the means ± SD (n = 6). *= p < 0.05 versus control, a = p < 0.05 versus oxysterols treated cells (Oxy).

**Fig. 2:**
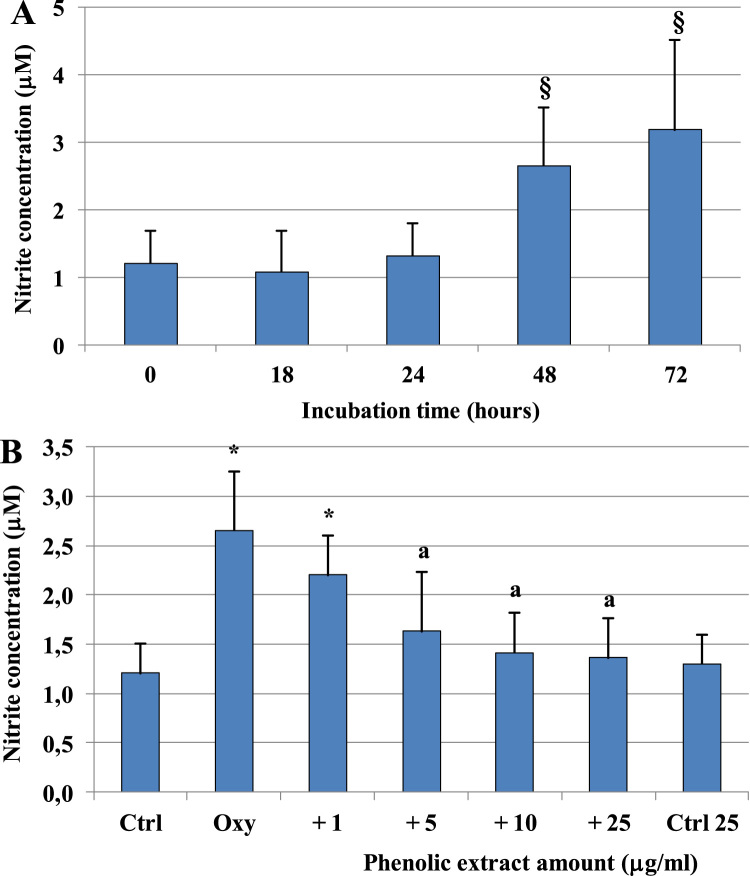
Lowering effect of the phenolic extract on oxysterols-induced NO release. NO levels measured as total nitrite concentration in differentiated Caco-2 cells treated with 60 μM oxysterol mixture for different incubation times (A) or pretreated (30 min) with different phenolic extract concentrations (1–25 μg/ml) and incubated in the presence of 60 μM oxysterol mixture for 48 h (B). NO levels were also evaluated in cells treated only with the phenolic extract 25 μg/ml (Ctrl 25). Values represent the means ± SD (n = 9). § = p < 0.05 versus time 0; *= p < 0.05 versus control, a = p < 0.05 versus oxysterols treated cells (Oxy).

Treatment of Caco-2 cells with the oxysterol mixture 60 µM for 24 h induced a significant rise of the secretion of both interleukins, doubled in the case of IL-8 and increased by about tenfold for IL-6, as shown in Fig. 1. Pre-treatment for 30 min with the phenolic extract, 25 µg/ml, a concentration that did not exert any cytotoxic effect (data not shown), significantly reduced the amount of interleukins detected in the culture medium. Oxysterols mixture was also able to induce the release of another key inflammatory mediator, NO (Fig. 2). A time-dependent increase of NO levels was found in the culture medium of oxysterols treated cells, showing a significant difference compared to control starting from 48 h of incubation. At all the tested concentrations the phenolic extract was able to inhibit NO release at 48 h. Phenolic extract showed no significant differences compared to control group starting from 5 μg/ml concentration.

### 3.2 Olive oil phenolic extract attenuates oxysterols -induced redox imbalance

**Fig. 3:**
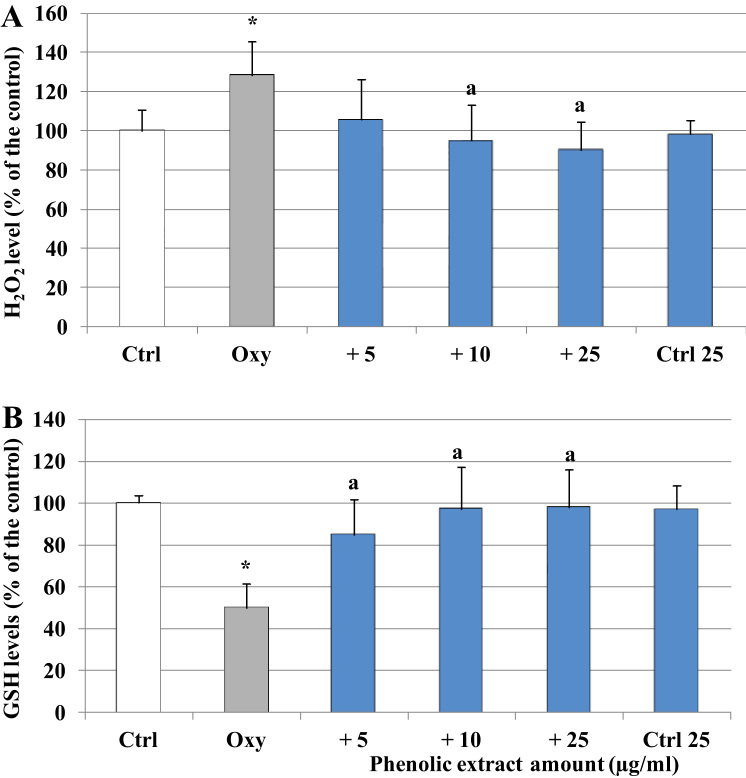
Modulation of cell H_2_O_2_ production and GSH levels by oxysterols and phenolic extract. A) H_2_O_2_ levels were determined in differentiated Caco-2 cells pretreated or not with different phenolic extract concentrations (5–25 μg/ml) for 30 min and incubated for 1 h in presence of 60 μM oxysterol mixture. Cells were further exposed to 2′,7′-dichlorodihydrofluorescein diacetate H_2_-DCF-DA (10 μM) for 30 min. B) GSH cell levels were measured in differentiated Caco-2 cells pretreated or not with different phenolic extract concentrations (5–25 μg/ml) for 30 min and incubated in the presence of 60 μM oxysterol mixture for 1 h. Both H_2_O_2_ and GSH levels were also evaluated in cells treated only with the phenolic extract 25 μg/ml (Ctrl 25). Values, expressed as percentage of the controls (GSH control value = 28 μM), represent the means ± SD (n = 9). * = p < 0.05 versus control, a = p < 0.05 versus oxysterols treated cells (Oxy).

The effect of the oxysterol mixture and the phenolic extract was then investigated in relation to the modulation of the cellular redox status. After 1 h of incubation a significant production of oxidant species associated with a dramatic reduction of cellular GSH, half of the amount detected in the control samples, was observed in the cells treated with the oxysterol mixture (Fig. 3). The presence of the phenolic extract in the reaction environment completely inhibited the formation of oxidant species and preserved the initial concentration of GSH.

### 3.3 Olive oil phenolic extract modulates the main cellular pathways activated in response to oxysterols treatment

**Fig. 4:**
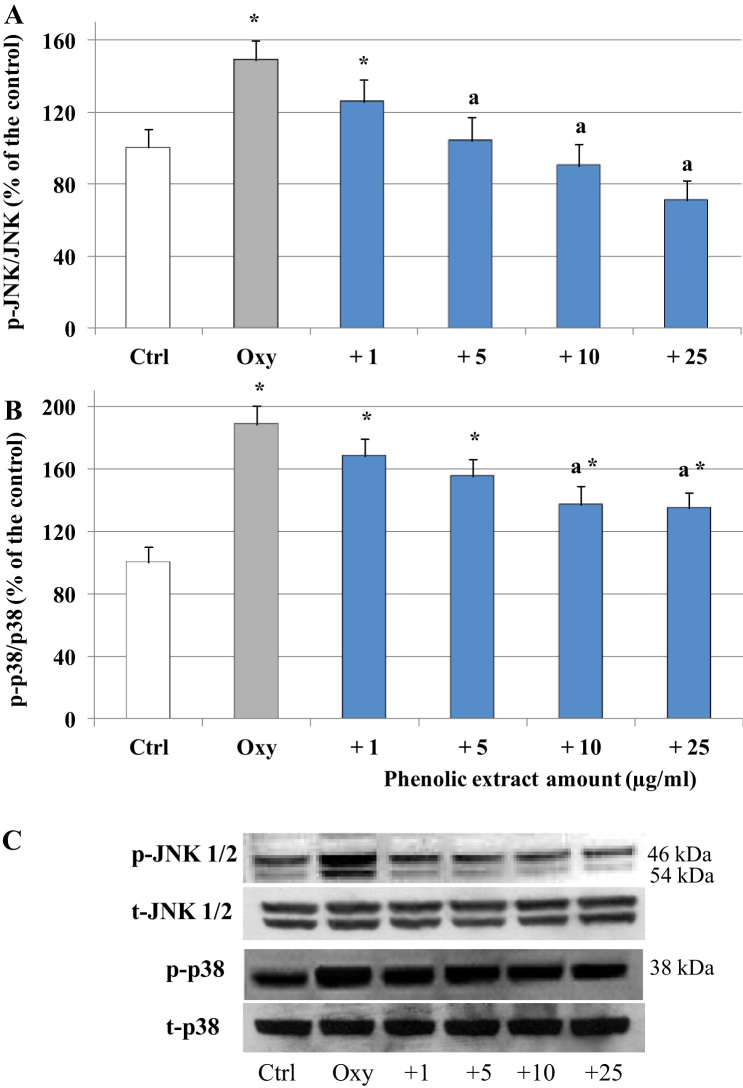
Modulation of JNK 1/2 and p38 phosphorylation by oxysterols and phenolic extract. JNK 1/2 (A) and p38 (B) activation in differentiated Caco-2 cells pretreated or not with different phenolic extract concentrations (1–25 μg/ml) for 30 min and incubated in the presence of 60 μM oxysterol mixture for 1 h. Values, expressed as percentage of the controls, represent the means ± SD (n = 4). *= p < 0.05 versus control, a = p < 0.05 versus oxysterols treated cells (Oxy). Representative WB of the treatment is shown (C).

**Fig. 5:**
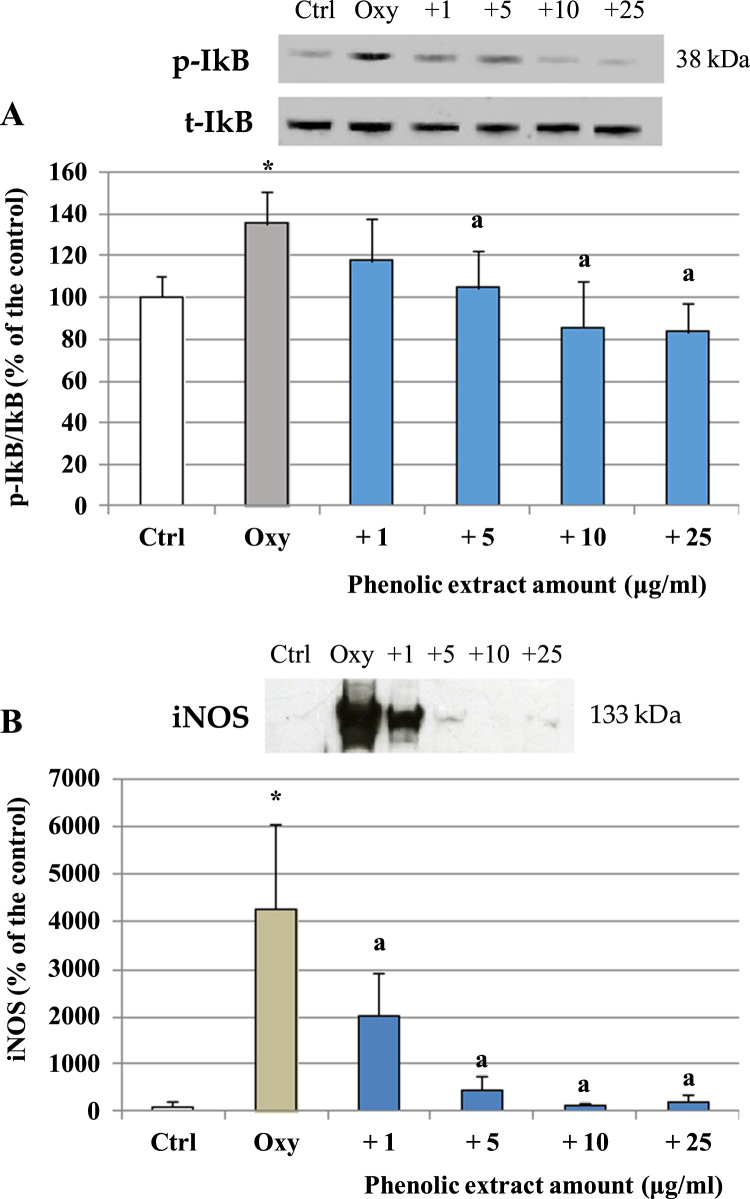
Modulation of IkB phosphorylation and iNOS levels by oxysterols and phenolic extract. A) IkB phosphorylation in differentiated Caco-2 cells pretreated or not with different phenolic extract concentrations (1–25 μg/ml) for 30 min and incubated in the presence of 60 μM oxysterol mixture for 6 h; B) iNOS level in differentiated Caco-2 cells pretreated or not with different phenolic extract concentrations (1–25 μg/ml) for 30 min and incubated in the presence of 60 μM oxysterol mixture for 48 h. Values, expressed as percentage of the controls, represent the means ± SD (n = 4). * = p < 0.05 versus control, a = p < 0.05 versus oxysterols treated (Oxy). Representative WB of the treatment (after IP in the case of iNOS) is shown.

The ability of the phenolic extract to modulate oxysterols interference with intracellular signaling pathways involved in enterocytes pro-inflammatory response was evaluated, focusing in particular on the MAPKs JNK 1/2 and p38. Caco-2 cells were incubated with the oxysterols mixtures for 1 h, after pre-incubation with the phenolic extract (30 min). As shown in Fig. 4A, oxysterols induced a significant increase of JNK 1/2 phosphorylation compared to control, which, on the contrary, was not observed in presence of the phenolic extract. Band values were normalized using the corresponding values of total proteins, and expressed as a percentage of the control. Similarly, after 1 h oxysterols treatment p38 phosphorylation was also induced. Phenolic extract pretreatment was able to decrease, even not completely, p38 induction (Fig. 4B). To further explore the molecular mechanism underlying the anti-inflammatory effect of the phenolic extract, we determined its role in NF-kB activation, by investigating as indirect marker IkB degradation. Western blot analysis showed that 60 μM oxysterol mixture produced a significant IkB phosphorylation after 6 h treatment, which is consistent with an up-regulation of NF-kB binding activity (Fig. 5) and again, the phenolic extract reverted oxysterols effect. Western blot analysis also revealed an increase of iNOS concentration after 48 h of oxysterols treatment with respect to the control level (Fig. 5) that was inhibited in the cells pretreated with the phenolic extract, reaching control value at the higher concentrations tested.

## 4 Discussion

Dietary habits may reduce or increase the inflammatory process linked to intestinal unbalance and damage that may lead to IBD initiation and progression. The ingestion of cholesterol oxidized products has been recently considered among the risk factors associated to the pathogenesis of IBD, being able to alter intestinal epithelial cells homeostasis [10], [11], [31], [32] towards a pro-inflammatory phenotype. Our data support the hypothesis of a direct pro-inflammatory effect of dietary oxysterols on intestinal Caco-2 cells. As previously shown in the same experimental conditions, incubation with a mixture of oxysterols representative of a hyper-cholesterol diet induced a significant secretion of IL-6 and IL-8, two key mediators of intestinal inflammation. Among the cytokines released by epithelial cells IL-8 holds a pivotal role in the recruitment of T lymphocytes and neutrophils at the site of inflammation [33], and it is up-regulated in the colon of patients with IBD [34]; IL-6 stimulates neutrophils chemotaxis, it is associated with tissue destruction in the colon [35] and its serum level is correlated with disease severity [36].

In the present work, we give the first evidence that oxysterols treatment also stimulates Caco-2 cells to increase NO production; differentiated Caco-2 cells constitutively produce NO, whose basal level may be increased upon exposure to LPS or various cytokines [37], [38]. NO may promote, attenuate, or have little effect on gut inflammation and injury, depending on its concentration and on the redox environment of its production. The role of iNOS-derived NO is not fully understood yet, however its sustained overproduction is considered to promote gut inflammation [39], [40]; NO may contribute to the enhancement of microvascular permeability, to the modulation of COX-2-dependent production of prostaglandins [41], to the chemotaxis and activation of leucocytes [42], and it may also rapidly interact with certain reactive free radicals such as O_2_^•-^ to yield the ONOO^**-**^ oxidant species [43]. The enhanced production of iNOS-derived NO is one of the most consistent findings in both experimental and human IBD, together with oxidant species production, indicating that chronically inflamed intestinal and/or colonic tissue is subjected to significant oxidative stress [44]. Oxysterols have been shown to promote oxidative stress through the production of oxidant species mediated at least in part by NOX-1 [9], [11] that, depending on the concentration used, may lead to oxidative damage [28]. In the present study, we detected a significant production of oxidant species in Caco-2 cells treated with dietary oxysterols with respect to controls, giving further evidence of their strong pro-oxidant effect. Hydrogen peroxide increase, in particular, caused a drop of GSH levels by profoundly altering cellular redox balance. Cellular GSH levels have been shown to significantly decrease in experimental IBD both at the initial disease stage [44] and when signs of inflammation are evident [45]. Oxidized glutathione (GSSG) was found highly increased compared with normal tissue in the inflamed mucosa from patients with active IBD [46]. Dietary oxysterols determine oxidant species overproduction and GSH levels imbalance thus likely playing a role in the early stages of IBD.

Antioxidant supplementation may prove beneficial if able to maintain the cellular redox balance, directly related to both oxidative damage and the modulation of redox sensible signaling pathways activated in the onset and first stages of the inflammatory process.

In this contest, we wanted to investigate the potential role of olive oil polyphenols in preventing the oxysterols -induced redox imbalance and pro-inflammatory phenotype in intestinal cells, as they have recently been addressed as potential beneficial agents in IBD [23], [24], [25]. Ingested polyphenols are poorly absorbed; most remains in the intestinal lumen, mainly in the colon, where may be particularly concentrated [47], [48], thus being able to act as direct antioxidants, scavenging oxidant species or preventing their formation [49]. Our data demonstrate that olive oil polyphenols are able to inhibit H_2_O_2_ and NO production triggered by oxysterols and preserve cellular GSH levels. The phenolic fraction extracted from extra virgin olive oil produced from Bosana variety has been shown to have a particular high content of phenolic compounds, mainly secoiridoids and active hydroxytyrosol, tyrosol and oleuropein [28], [50], and to exert a significant antioxidant activity in vitro, correlated to the quantity and quality of the phenolic content [28]. Phenols present in the extract may act through their simple direct antioxidant effect as free-radical scavengers, but also indirectly interfering with specific signaling proteins, modulated in response to oxidative stress and pro-inflammatory stimuli.

MAPK-NF-kB pathway is of particular interest among the intracellular pathways activated in IBD, because it seems to play a crucial role during intestinal inflammatory responses [51]. NF-kB takes part controlling the activation of various pro-inflammatory genes, leading to the production of pro-inflammatory cytokines such as IL-6 and IL-8 and to the expression of important inflammatory proteins such as cyclooxygenase-2 (COX-2) and iNOS [52]. In previous studies in differentiated Caco-2 cells we showed that oxysterols increased the amounts of p38 and JNK phosphorylated forms and activated NF-kB [15]. Consistently, in the experimental conditions of this study, we found that oxysterols significantly increased the phosphorylation of the two stress-activated MAPKs, p38 and JNK 1/2, and activate NF-kB, as shown by the phosphorylation of its inhibitor IkB. p38 phosphorylation is increased significantly in IBD tissue [53], [54] and in vitro and in vivo studies indicate that p38 activation mediates, among several downstream events induced in immune and non-immune cells [55], the production of IL-6 and IL-8 via NF-kB activation [56], [57]. Like p38, phosphorylated JNK1/2 are found highly concentrated in inflamed tissue from IBD patients [54], [58], it seems to contribute to the maturation and activation of immune cells and synthesis of different pro-inflammatory cytokines, however its role in IBD is controversial [59], [60].

Olive oil phenolic fraction has been shown to interact with MAPKs in LPS stimulated murine peritoneal macrophages, where a reduction of JNK and p38 phosphorylation resulted in an anti-inflammatory effect, blocking NF-kB activation [61]. In our Caco-2 cell cultures the phenolic extract was able to revert the oxysterols driven activation of JNK and p38 and subsequent phosphorylation of IkB. The inhibition of NF-kB activation may explain the lower concentration of Il-6 and Il-8 detected in the medium after oxysterols stimulation in presence of the phenolic extract. NF-kB is also involved in NO production via iNOS expression [52]. In the present study we found that exposure of Caco-2 cells to oxysterols resulted in a significant increase of NO production due to an upregulation of iNOS concentration, and treatment with the phenolic extract inhibited these effects.

The efficacy of the phenolic extract may be due to a possible synergic effect among the different compounds present in the same fraction, considering that some of them have been proven to interact with MAPKs and NF-kB signaling pathway. A few studies in vitro showed the ability of hydroxytyrosol to block NF-kB activation and iNOS and COX-2 expressions [61], [62], [63]. *In vivo* studies also indicate an anti-inflammatory effect of hydroxytyrosol and oleuropein exerted through the modulation of MAPKs signaling [23], [64].

Taken together our data clearly show that intestinal epithelial cells represent a direct target of the action of dietary oxysterols, giving further evidence of their possible implication in IBD development. Our results also suggest a significant protective effect at intestinal level of extra virgin olive oil polyphenols, strengthening the link between diet and IBD pathogenesis and progression. The inhibitory effects of olive oil total phenolic fraction on some key inflammatory mechanisms such as NF-kB activation, iNOS induction and IL-8 and IL-6 production, provide strong evidence that it is able to prevent or delay the progression of intestinal inflammation. Considering that also oleic acid, which represents the highly concentrated monounsaturated fatty acid in olive oil, has been proven to reduce colonic inflammation [65], extra virgin olive oil consumption may be considered a useful tool in the prevention and management of IBD.

## Funding

This work was supported by the RAS, Autonomous Region of Sardinia [LR7- CRP-25300; Annualità 2010].
